# Hemorrhagic Encephalopathy From Acute Baking Soda Ingestion

**DOI:** 10.5811/westjem2016.6.30713

**Published:** 2016-07-21

**Authors:** Adrienne Hughes, Alisha Brown, Matthew Valento

**Affiliations:** University of Washington, Division of Emergency Medicine, Seattle, Washington

## Abstract

Baking soda is a readily available household product composed of sodium bicarbonate. It can be used as a home remedy to treat dyspepsia. If used in excessive amounts, baking soda has the potential to cause a variety of serious metabolic abnormalities. We believe this is the first reported case of hemorrhagic encephalopathy induced by baking soda ingestion. Healthcare providers should be aware of the dangers of baking soda misuse and the associated adverse effects.

## INTRODUCTION

Baking soda is marketed to consumers for numerous household and personal purposes. Its active ingredient is sodium bicarbonate. Despite the widespread use of proton pump inhibitors and H2 blockers, baking soda continues to be used as an antacid for relief of indigestion. The recommended dosage for using baking soda as an antacid is ½ teaspoon in 4–8oz of water every two hours. Each teaspoon of baking soda contains 41.8mEq of sodium.^1^ Sodium bicarbonate is generally safe when used appropriately. However, if misused, it has the potential for significant toxicity. Metabolic alkalosis, hypernatremia, hypokalemia, hypochloremia, and hypoxia have been reported.^2^ Severe hypernatremia can cause neuronal cell shrinkage, retraction of cerebral tissue, and potentially intracranial hemorrhage. We present a case of severe metabolic alkalosis and hypernatremic hemorrhagic encephalopathy after an acute intentional baking soda ingestion.

## CASE REPORT

A 33-year-old male with a history of schizophrenia and polysubstance abuse presented to the emergency department (ED) with altered mental status. Emergency medical technicians reported that the patient was discovered in the middle of the street, agitated and confused with an empty box of baking soda in his pants pocket.

On initial evaluation, the patient appeared alert, tremulous and distressed. His vitals were temperature 35.7°C (96.2°F), heart rate 124 beats/min, respirations 18 breaths/min, blood pressure 126/93, oxygen saturation 94% on room air. The physical examination was significant for a thin male, rocking back and forth, mumbling incoherently and forcefully blinking his eyes. The head and neck examination was notable for horizontal nystagmus, intermittent involuntary facial twitching, moist mucus membranes and no facial droop. Pupils were equal, round, and reactive to light bilaterally. The cardiac examination revealed regular tachycardia. Neurologic examination was significant for a coarse tremor to his arms and upper torso. He would intermittently lift his legs and arms off the bed then slam them down on the stretcher. He was stuttering, disoriented, and unable to answer questions. Cerebellar function could not be tested due to the patient’s mental status. The rest of his exam was normal.

Initial laboratory values were Na 172mEq/L, K 2.5mEq/L, chloride 98mEq/L, CO_2_>45mEq/L, glucose 433mg/dL, BUN 16mg/dL, creatinine 1.85mg/dL, magnesium 3.2mg/dL, phosphate<1mg/dL, and calcium of 11mg/dL. Liver function tests were remarkable for a bilirubin of 1.4mg/dL, total protein of 8.5g/dL, albumin of 5.6g/dL. White blood cell count was 11.6 cells/microL and his hemoglobin was 17g/dL. A room air venous blood gas measurement 7.53, pCO_2_ 60mmHg, pO2 39mmHg, HCO_3_ 50mEq/L, with a base excess of 21.6mEq/L. The electrocardiogram (EKG) showed sinus tachycardia with a prolonged QTc of 528msec. Urinalysis: pH of 8.52 and granular casts. Serum osmolality was 364 mOsm/kg and venous lactate 12.3 mmol/L. A urine toxicology screen was negative for amphetamines, barbiturates, benzodiazepines, cocaine, methadone, opiates, phencyclidine, cannabionoids, and tricyclic antidepressants. Blood alcohol, acetaminophen, and salicylate levels were negative. A head computed tomography (CT) was obtained and revealed multiple areas of intracranial hemorrhage in the left temporal and bilateral cerebellar regions. Additionally there was subarachnoid hemorrhage in the left frontal lobe and right posterior frontal lobe (Figure). CT angiography was normal without aneurysm. CT of the chest, abdomen, and pelvis was significant only for diffuse mild dilation of the small bowel and marked fluid content of the entire GI tract.

While in the ED, the patient received 2L normal saline and intravenous potassium replacement. Neurosurgery was consulted but did not recommend any surgical intervention. He was admitted to the intensive care unit where he continued to receive IV normal saline and electrolyte repletion. The patient’s mental status improved to his baseline over the next 24 hours and he was able to endorse that he had consumed an entire box of baking soda (net wt 16oz/454g). His estimated sodium burden was 5,403mEq. A repeat head CT showed stable intracranial hemorrhages. He denied suicidal ideation and after evaluation, psychiatry deemed the patient safe for discharge. He was discharged on hospital day 4 at which time he had a non-focal neurologic exam.

## DISCUSSION

### Pathophysiology

Baking soda toxicity causes hypernatremic metabolic alkalosis with associated hypochloremia, hypokalemia and urinary alkalization, all of which were present in our patient.^3^–^7^ Healthy adult subjects less than 60 years old with normal renal function can tolerate up to 1700mEq of sodium bicarbonate with minimal symptoms.^8^ Our patient ingested an estimated 5,403mEq of sodium. Increased serum bicarbonate following ingestion results in increased renal excretion, known as bicarbonate diuresis.^3^,^9^,^10^ Such diuresis is accompanied by loss of chloride, sodium, potassium, and water. However, in large ingestions there can be substantial free water loss, resulting in an impaired glomerular filtration rate (GFR) and ultimately reduced bicarbonate filtering.

Central nervous system (CNS) hypernatremia causes an osmotic shift of water out of neurons, cellular dehydration and neuronal cell shrinkage. Cerebral volume loss causes retraction of tissues within the skull. Increased tension on dural bridging veins results in rupture of vascular structures and ultimately intracranial hemorrhage, most often in the subdural space.^11^

Certain populations are at greater risk of complications associated with baking soda misuse, including alcoholics (due to volume depletion associated with poor oral intake chronic vomiting), the elderly, hypovolemic patients, and patients with underlying pulmonary or renal disease.^12^

### Reasons for overdose

Published cases of baking soda toxicity frequently involve its excessive use as an antacid. 2,6,12–14 A retrospective review of all symptomatic cases reported to the California Poison Control system between 2000–2012 found that the most common reasons for reports were antacid misuse (60.4%), attempts to alter urine drug testing (11.5%), and urinary tract infection treatment (4.7%).^12^ There have been other reports of baking soda toxicity in the setting of pica and topical use in an infant with diaper rash.^3^,^15^ Our patient was unable to articulate his reasons for ingestion.

### Clinical Presentation

Baking soda toxicity can present in numerous ways. Most commonly, patients present with nausea, vomiting, and abdominal pain;^16^ however, 1–5% of patients will present with neurologic symptoms such as lethargy, drowsiness, nystagmus, seizures, weakness and rarely coma.^12^ Cardiac arrhythmias and cardiopulmonary arrest have been reported, as well as a case of a pregnant woman at 37 weeks gestation with baking soda pica who presented with rhabdomyolysis and peripartum cardiomyopathy.^5^,^15^,^17^ Spontaneous rupture of the stomach after sodium bicarbonate ingestion, thought to be due to increased CO^2^ production following bicarbonate reaction with acidic gastric contents, has also been reported.^18^

Published reports of hypernatremia as a cause of cerebral hemorrhage are rare. We could not find any cases of baking soda ingestion resulting in cerebral hemorrhage and hypernatremia. There are cases of hypernatremic hemorrhagic encephalopathy in neonates and children, typically associated with dehydration and/or medical treatments. Such cases usually have sodium levels over 160mmol/L.^19^–^22^ The first case of hypernatremic hemorrhagic encephalopathy in an adult patient was reported in 2010, the result of hypotonic fluid loss-induced hypernatremia.^11^

### Evaluation

Laboratory evaluation for suspected baking soda ingestion should include a complete metabolic panel, arterial blood gas and EKG.

Metabolic alkalosis, if severe, can lead to compensatory hypoventilation and hypercapnia, which was seen with our patient. Severe metabolic alkalosis is associated with a leftward shift of the oxygen-hemoglobin dissociation curve, resulting in impaired oxygen delivery to tissue and hypoxemia. Cardiopulmonary arrest and death from hypercapnic respiratory failure in the setting of metabolic alkalosis has been reported.^5^

Hypokalemia can result in a prolonged QT interval and subsequent ventricular arrhythmias.^5^,^6^

### Treatment

Treatment of toxic baking soda ingestions includes intravenous fluid resuscitation and potassium supplementation. Most cases of metabolic alkalosis will resolve with volume resuscitation. The first step is to calculate the free water deficit. The equation is as follows: FW Deficit = 0.6 x weight (kg) x (current Na mml/L / 140–1). The correction factor is 0.6 for men and children, 0.5 for women and elderly men, and 0.45 for elderly women. In acute settings (development of hypernatremia in minutes to hours) correction can occur with rapid transfusion of 5% dextrose in water with goal 1meq/L/hr. Dialysis can also be considered. In subacute settings (occurring over 1–2 days) correction can occur between 0.5–1meq/L/hr. In chronic settings (>2 days) correction should occur 0.5meq/L/hr for adults and 0.3meq/L/hr for pediatric patients to limit potential risk of cerebral edema that may be associated with rehydration.^23^,^24^ Unlike hyponatremia, rapid correction of hypernatremia in adults is not known to be harmful in the acute and subacute setting and many patients are under corrected.^23^,^25^ For pediatric patients with an unknown duration of hypernatremia, it is recommended that patients avoid correction>0.5meq/L/hr. Large potassium deficits are typically present with baking soda toxicity and should be aggressively monitored and replaced. Hemodialysis can be considered in critically ill patients with renal failure and severe electrolyte derangements that are not responding to fluid and electrolyte repletion.^26^

In the setting of ventricular dysrhythmias, electrolytes should be corrected. Amiodarone and lidocaine are first line for treatment of ventricular tachycardia.. Seizures should be treated with benzodiazepines.^8^

In summary, we present a case of a baking soda overdose resulting in hemorrhagic encephalopathy, metabolic alkalosis and hypernatremia. Baking soda can lead to life-threatening complications when misused. Healthcare providers should be aware of the common practice of using baking soda as a home remedy for indigestion and the potential dangers associated with its misuse. The combination of hypernatremia and metabolic alkalosis should raise suspicion for ingestion of baking soda. Although rare, severe acute hypernatremia can result in intracranial hemorrhage due to CNS dehydration and stretching of vascular structures within the skull.

## Figures and Tables

**Figure A, B and C:**
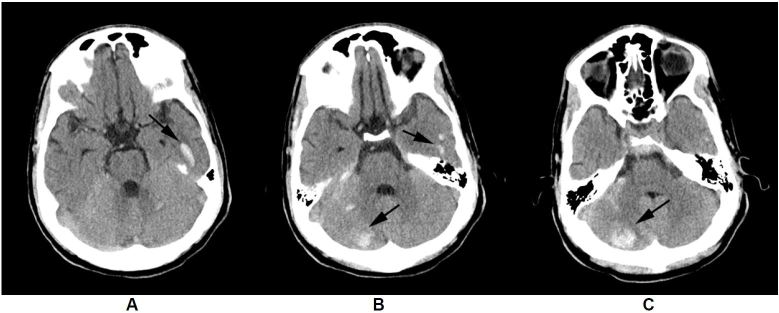
Non contrast CT head demonstrating left temporal and right cerebellar hemorrhages.
